# The Mediating Effect of Depression on the Relationship between Social Support, Spirituality and Burnout in Family Members of Patients with Cancer

**DOI:** 10.3390/ijerph18041727

**Published:** 2021-02-10

**Authors:** Won-Hee Jun, Kyung-Sook Cha, Kee-Lyong Lee

**Affiliations:** 1 College of Nursing, Keimyung University, Daegu 42601, Korea; jwh9178@hanmail.net; 2 Department of Nursing Science, Sun Moon University, Asan-si 31460, Korea; 3 Department of Nursing, Suwon Science College, Hwaseong-si 18516, Korea; ew10ew@hanmail.net

**Keywords:** burnout, cancer, depression, family, nursing, spirituality, support

## Abstract

When the treatment process of cancer patients changes to outpatient treatment, the burden on family members increases and they often experience burnout. Burnout not only effects the family members themselves but may also have a negative effect on the health of the cancer patient. Therefore, healthcare providers should evaluate burnout in the family members of cancer patients and actively make efforts towards their burnout management. This study investigated the mediating effect of depression on the relationship between social support, spirituality, and burnout in family members of patients with cancer. Participants were 151 family members of patients with cancer who were receiving chemotherapy as outpatients at a single university hospital in Korea. Data was collected from 2 March to 31 May 2016, using self-reported questionnaires. Collected data was analyzed with t-tests, analysis of variance (ANOVA), Pearson’s correlations, Baron and Kenny’s three-step regression method, and the Sobel test. The participants’ mean burnout was below the median. The participants’ depression partially mediated the relationship of both social support and spirituality on burnout. Strategies to assess depression and strengthen social support and spirituality should be developed to manage burnout in family members’.

## 1. Introduction

Regardless of the country, cancer directly and indirectly changes the lives of not only patients but also families [1,2]. When the treatment process for patients with cancer changes from typical inpatient treatment to outpatient treatment, patients with cancer spend more time at home [1,3]. The familial values in Korean society cause patient care to become their family members’ responsibility, which comes with an increased burden [4]. This burden can cause family members to fail to take care of their own health, which leads to increased psychological stress, physical discomfort, fatigue, and an increased risk of experiencing burnout [1,5,6]. Burnout can cause physical [7] and psychological problems [8,9], which reduces the quality of care provided to the patient and negatively effects the patients’ wellbeing and health [1,10]. However, most studies on the burnout of caregivers in Korea and overseas have focused on healthcare providers; therefore, it is necessary to evaluate the degree of burnout among family members of patients with cancer and identify the factors that influence burnout.

Korea is experiencing an increase in the average life expectancy due to economic development and improved healthcare, while the incidence of patients with cancer is also increasing due to changes in lifestyle and the environment [11]. However, the survival rate and the survival time of patients with cancer is steadily increasing with early diagnosis and improved treatment methods, and cancer is no longer an incurable disease—rather it is becoming a chronic disease that requires continuous management [2].

From the moment a patient is diagnosed with cancer, their family members perform various roles including physical nursing, psychological nursing, financial support, and housework [12]. Confucian culture, which emphasizes the practice of “filial piety”, is prevalent in Korean society. To match these social expectations, families must persevere during the treatment period [4,13]. However, because of the changes to daily life, burnout is easily experienced [14]. In studies conducted in Korea, the degree of burnout in family members of patients with cancer was moderate at 49.47 out of 100 [5], and a study conducted among parents of pediatric patients with cancer in Iran [15] reported the degree of burnout to be more than moderate at 4.26 out of 7. Consequently, healthcare professionals need to assess burnout in family members of patients with cancer and investigate relevant factors to actively intervene in their burnout management.

Burnout can be affected by internal and environmental factors [16]. Several factors that influence the burnout of caregivers have been reported, such as stress [1,5], social support [17,18], spirituality [19,20], health status [1], and depression [9,21]. Thus, there is an increasing interest in the effects of protective factors such as positive psychological characteristics (e.g., spirituality and social support) on caregivers’ burnout.

Social support has been reported to play a key role in alleviating physical and psychological health problems due to the stress of daily life and in promoting psychological wellbeing [22,23]. Social support has been reported as the most important predictor of burden in family members of patients with cancer [24]. Further, the higher the level of perceived social support, the lower the burnout in family members of patients with dementia [18] or chronic diseases, such as multiple sclerosis and Alzheimer’s disease [17].

Spirituality is an internal resource that allows humans to cope with stress and re-establish positive values even in negative circumstances [25], and it can have a positive effect on maintaining and promoting health in stressful situations [26]. According to a study conducted in Korea among healthcare providers [19,20], the degree of burnout was lower when spirituality was high, and spiritual wellbeing was a major influencing factor of burnout [27]. Further, in a study from Oman, which was conducted among expatriate lecturers, the degree of burnout was lower among participants who reported high spirituality [28]. Further, burnout in parents of pediatric patients with cancer, who were provided with a spiritual intervention program, significantly decreased [15]. Therefore, the spirituality of family members of patients with cancer can be presumed to be a predictor that reduces burnout; however, globally, there has not been any direct research on the relationship between spirituality and burnout in family members of patients with cancer.

Furthermore, the most common emotional problem experienced by family members of patients with cancer is depression [29]. Depression is an emotional reaction in the process of burnout. It is not only a cause of burnout but also a major factor in aggravating it [30]. However, depression can be controlled by social support or spirituality. In a study of family members of patients with Alzheimer’s disease, social support was negatively correlated with depression in those family members [31]. The level of depression was also lower in family members of patients with cancer when those family members felt they were supported [32]. Moreover, Green [33] suggested that spirituality was closely related with depression in family members of patients with Alzheimer’s disease, and a study by Koenig et al. [34] on inpatients also reported spiritual experience to have a positive effect on preventing depression. The results of these previous studies suggest that depression can play a mediating role in the relationships among social support, spirituality, and burnout.

Although there have been studies investigating the direct effects of depression on burnout, no studies have examined the relationships between the variables, including social support and spirituality, which may be protective factors for depression and burnout. Given the recent tendency to focus on strengthening protective factors when intervening in health problems such as burnout [15,28,35], if the role of depression is clarified—in regard to its association with social support, spirituality, and burnout in family members of patients with cancer—it could inform a more concrete intervention plan for burnout management.

## 2. Materials and Methods

### 2.1. Aims

The aims of this study were as follows. First, we determined the how the prevalence of burnout correlates with the general characteristics of family members of patients with cancer. Second, we identified the mediating effect of depression on the relationship between social support and burnout in family members of patients with cancer. Third, we identified the mediating effect of depression on the relationship between spirituality and burnout in family members of patients with cancer.

### 2.2. Design

This research employed a cross-sectional, descriptive design.

### 2.3. Participants

The participants were the family members of patients with cancer undergoing outpatient chemotherapy at one university hospital in Sejong city, Korea. They were conveniently sampled. The inclusion criteria were adults aged 20–79 years without a history of a cognitive or mental illness diagnosis/treatment, who understood the content and purpose of the study, as well as provided written consent to participate.

The number of participants was estimated using G-power 3.1.5. With the significance level set at 0.05, a medium effect size of 0.15, a statistical power of 80%, and 13 predictive factors (10 general characteristics and 3 independent variables). The number of participants needed for a multiple regression analysis was 131. Allowing for a dropout rate of 20%, we surveyed 160 people.

### 2.4. Measures

The participants’ general characteristics that we measured were age, sex, education, religion, occupation, financial burden, relationship to patient, number of family caregivers, recurrence, and patients’ activities of daily living.

The burnout measurement tool we used was developed by Pines et al. [36] and translated into Korean by Peek [37]. It consisted of 20 items: 6 for physical burnout, 7 for emotional burnout, and 7 for psychological burnout. Each item was measured on a 5-point Likert scale ranging from “strongly disagree” to “strongly agree.” The higher the score, the higher the experience of burnout. In studies by Peek [37] and Hong and Tae [1], who examined family members of patients with cancer, Cronbach’s αs for the reliability of the tool were 0.86 and 0.89, respectively. Cronbach’s α was 0.93 in the current study.

The social support measurement tool we used was developed by Park [38]. It consisted of 25 items: 9 for emotional support, 6 for material support, 6 for appraisal support, and 4 for information support. Each item was measured on a 5-point Likert scale ranging from “strongly disagree” to “strongly agree.” Higher scores indicated a higher degree of social support. Cronbach’s αs for the reliability of the tool were 0.93 in Park [38], 0.97 in Hong and Tae [1], and 0.97 in the current study.

The spirituality measurement tool we used was developed by Lee et al. [39] for the Korean population. It consisted of 30 items across 6 subcategories—transcendence, meaning and purpose of life, compassion, inner resource, awareness, and connectivity—each comprising 5 items. Each item was measured on a 5-point Likert scale ranging from “strongly disagree” to “strongly agree”, and the higher the score, the higher the spirituality. Cronbach’s αs for the reliability of the tool at the time of the development and in the current study were 0.93 and 0.96, respectively.

The depression measurement tool we used was the Center for Epidemiologic Studies Depression Scale, which was developed by Radloff [40] and translated into Korean by Chon et al. [41]. It consisted of 20 items, and each item was measured on a 4-point Likert scale ranging from 0 (“rarely”) to 3 (“most of the time”). The higher the score, the higher the severity of depression, and a total score higher than 16 points indicated clinical depression [41]. Cronbach’s αs for the reliability of the tool were 0.91 in Chon et al. [41] and 0.83 in the current study.

### 2.5. Data Collection

Data was collected from 2 March 2016 to 31 May 2016 using self-reported questionnaires. A study investigator and research assistants who were trained in data collection visited the outpatient departments of internal medicine and surgery, as well as outpatient injection rooms of the university hospital that allowed data collection. Participants were informed of the study purpose and content directly. Questionnaires were completed independently and collected immediately. All 160 questionnaires were collected with 151 questionnaires included in data analyses. Nine questionnaires were missing data and excluded (valid response rate = 94.3%).

### 2.6. Ethical Considerations

This study was conducted after obtaining approval from the institutional review board and the department head of the involved institution. The investigator explained the study purpose, protection of personal information, anonymity, and statistical analysis techniques. Participants were also informed that the data would only be used for this study and that they could withdraw their participation at any time without consequence. All participants provided written consent. The survey took about 30 min to complete, and completed questionnaires were collected immediately.

### 2.7. Data Analysis

Collected data were analyzed using SPSS 25.0 (IBM Corp., Armonk, NY, USA). The means and standard deviations of the degree of social support, spirituality, depression, and burnout were identified. The difference in burnout according to the general characteristics of the participants was analyzed with t-tests and an analysis of variance. Scheffé’s test was used for post-hoc analysis. For the correlations between variables, Pearson’s correlations were used. To test the mediating effect of depression on the relationships among social support, spirituality, and burnout, we used a three-step simple and multiple regression analysis and the Sobel test, as suggested by Baron and Kenny [42].

The three conditions for establishing the mediating effect were as follows: in the first step of the regression analysis, the independent variable must have a significant influence on the mediator; in the second step of the regression analysis, the independent variable must have a significant influence on the dependent variable; in the third step of the regression analysis, the mediator must have a significant influence on the dependent variable, and the influence of the independent variable on the dependent variable must be reduced in the third step compared to the second step. If the relationship between the independent variable and the dependent variable is not significant in the third step then it is interpreted as full mediation, whereas it is interpreted as partial mediation if the relationship is significant.

### 2.8. Validity and Reliability

For all scales, Cronbach’s α values ranged from 0.83 to 0.97, indicating acceptable to excellent reliability. All measures used in this study had been previously validated with family members of patients with cancer or adult populations in Korea.

## 3. Results

### 3.1. Participants’ General Characteristics and Study Variables

Participants’ demographic characteristics are shown in Table 1.

### 3.2. Participants’ Degree of Burnout, Social Support, Spirituality, and Depression

The mean scores and correlations among study variables are shown in Table 2. Burnout was negatively associated with social support and spirituality, and it was positively associated with depression.

### 3.3. Participants’ Characteristics and Difference in burnout

There was a difference in burnout in participants based on their education, financial burden, number of family caregivers, recurrence, and patients’ ADL (Table 1). The post-hoc analysis revealed that the burnout score of the elementary graduate group was higher than that of the college or higher graduate group. Further, the greater the financial burden, the higher the burnout score. The burnout score of patients with recurrence was higher than that of patients without recurrence. Lastly, the burnout score of patients with very dependent ADL was higher than that of independent patients.

### 3.4. Mediating Effect of Depression on the Relationship among Social Support, Spirituality, and Burnout

**Figure 1:**
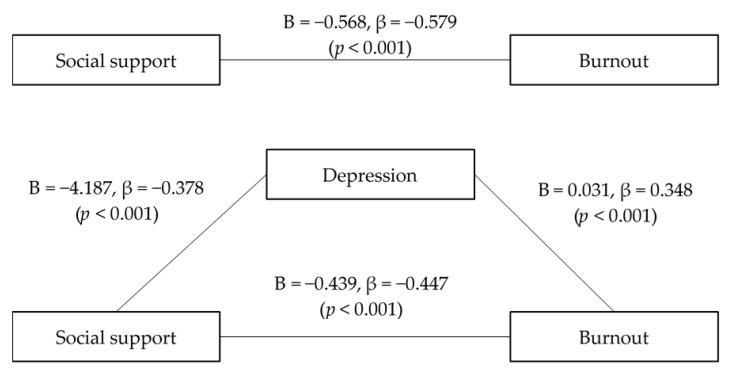
Mediation model of the effects of depression on the relationship between social support and burnout (Sobel’s test: Z = −3.589, *p* < 0.001).

Concerning social support, the first step of the regression analysis, which addressed the effect of social support (an independent variable) on depression (a mediator) was significant. The second step, which addressed the effect of social support (an independent variable) on burnout (a dependent variable) was significant. Lastly, the effect of depression (a mediator) on burnout was significant after controlling for social support (an independent variable). Here, the effect of social support (a dependent variable) on burnout was significant, and it showed a partial mediation of depression. Further, depression significantly mediated the relationship between social support and burnout (Figure 1).

**Figure 2:**
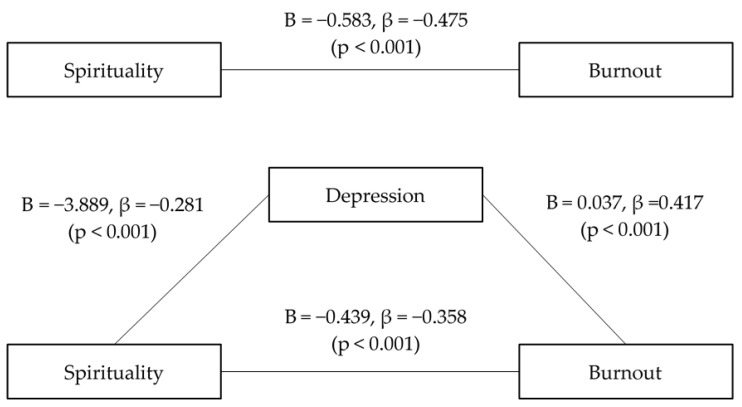
Mediation model of the effects of depression on the relationship between spirituality and burnout (Sobel’s test: Z = −3.092, *p* = 0.001).

Concerning spirituality, the first step of the regression analysis, which addressed the effect of spirituality (an independent variable) on depression (a mediator) was significant. The second step, which addressed the effect of spirituality (an independent variable) on burnout (a dependent variable) was significant. Lastly, the effect of depression (a mediator) on burnout was significant after controlling for spirituality (an independent variable). Here, the effect of spirituality (a dependent variable) on burnout was significant, and it showed a partial mediation of depression. Further, depression significantly mediated the relationship between spirituality and burnout (Figure 2).

## 4. Discussion

The purpose of this study was to identify the mediating effect of depression on the relationship of both social support and spirituality on burnout in family members of patients with cancer. Interventions to reduce burnout in family members of patients with cancer can be developed and introduced based on the results of this study.

In this study, the mean burnout in participants was below the median. Considering that recurrence of cancer increases caregivers’ burnout [5], our results imply that most participants did not have burnout because most patients had no cancer recurrence. In addition, the degree of burnout among participants was similar to the result of 2.43 points found by Hong and Tae [1], who used the same measurement tool to assess burnout among family members of patients with cancer. However, our participants experienced less burnout than those in a study of parents of pediatric patients with cancer (4.26 out of 7) [15] and intensive care unit (ICU) nurses (3.18 out of 5) [43]. Such differences may be related to the degree of patient dependence on the caregiver. The current participants were family members of adult patients with cancer who were receiving outpatient treatment; therefore, their degree of burnout may be lower than that of ICU nurses who care for patients with severe conditions and are fully dependent on the nurses for all activities, and the parents of children, who are more dependent on caregivers because of their physical and mental immaturity.

Participants’ burnout significantly differed with respect to education, number of family caregivers, financial burden, recurrence, and ADL. The degree of burnout was higher in the elementary graduate group than in the college or higher graduate group, which aligned with prior results [5,21]. Less education may result in an increased likelihood of burnout as it is difficult to cope with stress effectively due to the lack of ability to find and use help in stressful situations [24]. Therefore, when attempting educational interventions for the burnout in family members of patients with cancer, it is vital to consider their education level.

The burnout in participants was lower when the number of family caregivers was high and when the patient was independent. Considering the reality in Korea, where families are primarily responsible for caring for cancer patients [4], if there are many family members participating in care and patients can live their daily lives independently, the physical burden of patient care may be reduced and the time for leisure may be in-creased. This may result in less burnout [5].

Moreover, the degree of burnout was higher with high financial burden and with cancer recurrence, which also aligned with prior results [5]. In Korea, 95% of the treatment and medication costs are provided to cancer patients who have signed up for the national health insurance system, and most patients receive these benefits [44]. However, in the case of cancer patients, economic activities are limited, which can cause difficulties in raising funds for maintaining daily life in addition to paying for medical expenses. Moreover, cancer patients need years of long-term care and management, and they are at a high risk for recurrence, which is a stressor for families who care for them and can lead to burnout [45]. Therefore, we need policies that not only support cancer patients but also their families to maintain their daily lives and focus on treatment during the disease.

Depression may partially mediate the relationship between social support and burnout in family members; in other words, having a higher level of perceived social support may reduce burnout by decreasing depression. This aligned with the results of Karadavut and Uneri [46], who reported that family support reduced burnout and depression among mothers of patients with a brachial plexus injury, as well as Hong and Tae [1], who reported that social support influenced burnout with stress as a mediator, which is closely related to depression. In addition, the current results support previous studies that reported a negative correlation between social support and depression in spouses of patients with breast cancer [47], and a more serious degree of burnout with a high degree of depression in caregivers of patients with dementia [9].

Perceived social support fosters positive psychological characteristics—such as hope—to help individuals effectively cope with stress [1,48]. This can reduce depression by promoting psychological stability [47]. Therefore, family members of patients with cancer may need to utilize mental health professionals or clinical nurses as supportive resources, both emotionally and physically. Moreover, healthcare providers should make efforts to provide information about psychological support to volunteers or alternative caregivers and promote the use of self-help groups, all of which can assist family members of patients with cancer.

Depression partially mediated the relationship between spirituality and burnout. This aligned with the results of Ho et al. [19], who reported that depression and burnout was reduced with an increase in spiritual experience, and a study of medical students that reported a lower degree of burnout with high spirituality [49]. Moreover, our results support Green [33], who claimed the need for promoting spirituality of family members to reduce the frequency and intensity of depression among caregiving family members.

Individuals with spiritual wellbeing tend to display less emotional coping in stressful situations [35,50]. This reduces negative emotions such as depression or anxiety, and helps maintain psychological stability and internal peace, which have positive effects on burnout [43,50,51]. Recently, in clinical nursing settings, interest in spiritual care being included in holistic nursing care is increasing [52]. In this context, clinical nurses should develop programs to promote not only patients’ spirituality but also family caregivers’ spirituality to enhance their ability to cope and promote a sense of wellbeing [52]. In addition, these programs may be effective in evaluating the degree of depression in family caregivers and should include emotional regulation training.

The findings of this study may contribute to broadening the understanding of burnout in family members of patients with cancer. Our study has important implications for healthcare providers in clinical nursing and community settings, providing a useful guideline for healthcare providers when establishing practical and specific intervention strategies for managing burnout in family members of patients with cancer. We only collected data from one university hospital; therefore, there are limits to the generalizability of the current results. Further, because self-reporting questionnaires were used, the possibility of participants responding defensively or responding according to socially desired characteristics cannot be excluded.

## 5. Conclusions

Burnout in family members of patients with cancer not only has negative effects for their own health but it also compromises the health of the patients. It is necessary for healthcare providers to assess burnout in family members of patients with cancer and identify related factors to actively intervene in burnout management. We found that family members’ depression partially mediated the effects of both social support and spirituality on burnout. Therefore, to manage family members’ burnout, it may be effective to strengthen social support and spirituality, as well as assess for depression and intervene when necessary.

Based on our results, we have the following suggestions. First, it is necessary to validate our results through repeated research that extends the number of participants and regions. Second, a study should examine how to strengthen the spirituality and social support of family members of patients with cancer. Third, a program should be developed to that evaluate depression and the effects of burnout. Lastly, there should be a study about the potential behavioral mediators that impact burnout in family members of patients with cancer.

## Figures and Tables

**Table 1:** Participants’ demographic characteristics and differences in burnout (n = 151).

Characteristic	Category	n (%)	Burnout		
			Mean ± SD	F/t	*p*
Age (years)	<30	7 (4.6)	2.56 ± 0.91	1.37	0.246
	30–39	36 (23.8)	2.55 ± 0.70		
	40–49	44 (29.2)	2.48 ± 0.63		
	50–59	35 (23.2)	2.80 ± 0.58		
	≥60	29 (19.2)	2.71 ± 0.69		
Sex	Male	43 (28.5)	2.61 ± 0.77	0.09	0.932
	Female	108 (71.5)	2.62 ± 0.63		
Education	Elementary school ^a^	6 (4.1)	3.42 ± 0.96	4.61	0.004 a > b
	Middle school	15 (9.9)	2.54 ± 0.76		
	High school	57 (37.7)	2.72 ± 0.61		
	College or higher ^b^	73 (48.3)	2.49 ± 0.62		
Religion	Protestant	25 (16.6)	2.67 ± 0.57	1.65	0.179
	Catholic	51 (33.8)	2.46 ± 0.57		
	Buddhist	17 (11.3)	2.60 ± 0.70		
	None	58 (38.4)	2.74 ± 0.76		
Occupation	Yes	84 (55.6)	2.54 ± 0.63	1.52	0.131
	No	67 (44.4)	2.71 ± 0.71		
Financial burden	Easy ^a^	33 (21.9)	2.21 ± 0.68	15.56	<0.001 a < b < c
	Average ^b^	63 (41.7)	2.54 ± 0.59		
	Hard ^c^	55 (36.4)	2.94 ± 0.59		
Relationship to patient	Spouse	53 (35.1)	2.77 ± 0.64	1.94	0.106
	Parents	12 (7.9)	2.42 ± 0.58		
	Children	59 (39.1)	2.47 ± 0.75		
	Daughter-in-law	14 (9.3)	2.79 ± 0.35		
	Other	13 (8.6)	2.66 ± 0.59		
Number of family caregivers	1	35 (23.2)	2.87 ± 0.68	3.02	0.032
	2	42 (27.8)	2.65 ± 0.65		
	3	44 (29.1)	2.53 ± 0.65		
	≥4	30 (19.9)	2.41 ± 0.63		
Recurrence	Yes	43 (28.5)	2.79 ± 0.66	2.06	0.041
	No	108 (71.5)	2.55 ± 0.66		
Patients’ ADL	Very dependent	13 (8.6)	2.81 ± 0.70	3.95	0.021 a > b
	Little dependent ^a^	76 (50.3)	2.73 ± 0.60		
	Independent ^b^	62 (41.1)	2.44 ± 0.70		

ADL: activities of daily living. ^a, b, c^: Analyzed by Scheffé’s test.

**Table 2:** Correlations among study variables (n = 151).

Variable	Mean ± SD	Social Support	Depression	Spirituality
		r (*p*)		
Burnout	2.62 ± 0.67	−0.579 (<0.001)	0.517 (<0.001)	−0.475 (<0.001)
Social support	3.49 ± 0.68	1	−0.378 (<0.001)	0.578 (<0.001)
Depression	16.83 ± 7.53		1	−0.281 (<0.001)
Spirituality	3.35 ± 0.54			1

## Data Availability

The data used and/or analyzed during the current study are available from the corresponding author on request.
